# Validation of less-invasive hemodynamic monitoring with Pulsioflex in critically ill patients

**DOI:** 10.1186/cc10833

**Published:** 2012-03-20

**Authors:** M Peetermans, W Verlinden, J Jacobs, A Verrijcken, S Pilate, N Van Regenmortel, I De laet, K Schoonheydt, H Dits, ML Malbrain

**Affiliations:** 1 ZNA Stuivenberg, Antwerp, Belgium

## Introduction

Thermodilution (TD) is a gold standard for cardiac index (CI) measurement. The aim of this study is to compare intermittent bolus TDCI with intermittent automatic calibration CI (AutoCI) and continuous CI (CCI) obtained by pulse contour analysis with PiCCO_2 _(PiCCI) and Pulsioflex (PuCCI).

## Methods

A prospective study in 20 patients (all mechanically ventilated, 14 male). Age 54.4 ± 16.7, BMI 28.1 ± 7.3, SAPS II 52.9 ± 13.4, APACHE II score 26.7 ± 7.8 and SOFA score 10 ± 3. All patients underwent PiCCO monitoring via a femoral line whilst the radial line was kept in place during four 8-hour time periods (in the first two periods the Pulsioflex was connected to the radial line, in the last two it was connected to the femoral line). In the first and third 8-hour periods the Pulsioflex was calibrated with the TDCI obtained at baseline, for the second and fourth 8-hour periods the Pulsioflex was calibrated with the AutoCI value. Simultaneous PiCCI and PuCCI measurements were obtained every 2 hours while simultaneous TDCI and AutoCI were obtained every 8 hours. The PiCCI and PuCCI values were recorded within 5 minutes before TDCI was determined. We also looked at the effects of 22 interventions: passive leg raise (*n *= 6), fluid bolus (*n *= 5), change in vasopressor (*n *= 9) or dobutamine (*n *= 1), increase in sedation (*n *= 1). Statistical analysis was performed using Pearson correlation and Bland-Altman analysis.

## Results

In total, 305 paired PiCCI-PuCCI and 128 paired AutoCI-TDCI values were obtained. TDCI values ranged from 1.5 to 6.7 l/minute/m^2 ^(mean 3.9 ± 1), AutoCI from 2.4 to 6.5 (3.8 ± 0.8), PiCCI from 1.5 to 7.1 (3.8 ± 1.2) and PuCCI from 2 to 7.6 (3.8 ± 1). The Pearson correlation coefficient comparing all and mean PuCCI and PiCCI values had an *R*^2 ^of 0.77 and 0.86 respectively; for AutoCI and TDCI, *R*^2 ^was 0.76. The above *R*^2 ^values were 0.73, 0.84 and 0.71 respectively when the Pulsioflex was connected to a radial line. Changes in AutoCI correlated well with changes in TDCI (*R*^2 ^= 0.68), as did changes in PuCCI versus changes in PiCCI (*R*^2 ^= 0.53). PPV obtained from Pulsioflex and PiCCO correlated better than SVV (*R*^2 ^= 0.86 vs. 0.62). Changes in PiCCI and PuCCI induced by an intervention correlated well with each other (*R*^2 ^= 0.94). Bland-Altman analysis comparing AutoCI with TDCI revealed a mean bias ±2SD (LA) of 0.05 ± 0.94 l/minute/m^2 ^(with 27.3% error) while analysis of PuCCI versus PiCCI showed a bias ±LA of 0.01 ± 1.12 (29.1% error).

## Conclusion

Although TDCI remains a gold standard, the preliminary results of an ongoing prospective study indicate that in unstable critically ill patients CI can be reliably monitored with Pulsioflex technology. Moreover, the Pulsioflex was also able to keep track of changes in CI.

